# The Impact of Life and Adverse Childhood Events on Help-Seeking Behaviours—A Cross-Sectional Survey of School-Age Adolescents in Jordan

**DOI:** 10.3390/pediatric17010022

**Published:** 2025-02-10

**Authors:** Reham A. Lasheen, Sara Abu Khudair, Yousef Khader, Eizaburo Tanaka, Mohannad Al Nsour

**Affiliations:** 1 The Eastern Mediterranean Public Health Network, Amman 11196, Jordan; sara.a.khudair@gmail.com (S.A.K.); executive.director@emphnet.net (M.A.N.); 2 Department of Health Psychology, School of Population Health, Royal College of Surgeons in Ireland, D04 DH60 Dublin, Ireland; 3 Health Sciences Unit, Faculty of Social Sciences, Tampere University, 33520 Tampere, Finland; 4 Department of Community Medicine, Public Health and Family Medicine, Faculty of Medicine, Jordan University of Science and Technology, Irbid 22110, Jordan; 5 College of Arts and Sciences, Komaba Organization for Educational Excellence, The University of Tokyo, Tokyo 113-0033, Japan; eizaburo.tanaka.20@alumni.ucl.ac.uk

**Keywords:** traumatic life events, adverse childhood events, help-seeking behaviours, adolescents’ mental health

## Abstract

Traumatic life and childhood events are associated with adverse health outcomes, particularly for adolescents, who are vulnerable to such events and exhibit distinct health behaviours and needs. Nevertheless, the influence of exposure to these events on their help-seeking behaviour remains largely unexplored, especially in the Eastern Mediterranean region. This study aims to estimate the prevalence of adverse events among adolescents in Jordan and examine how adverse events shape the help-seeking behaviours. Methods: A national cross-sectional survey of 4407 school-age (12–18 years) adolescents living in Jordan was conducted between December 2022 and April 2023 using multi-state stratified cluster sampling. The study utilised self-report questionnaires as well as validated tools. These were adapted to ensure cultural relevance and sensitivity and translated to Arabic. Results: The prevalence of at least one adverse event is around 16%, while that of four or more ACEs stands at around 41% in our population. The most commonly reported event was being infected or having a family member infected with COVID-19 at 60.3%. Specific individual characteristics and traumatic events appeared to shape their help-seeking behaviour, particularly family affluence and smoking status as well as exposure to COVID-19. Conclusions: The study underscores the need to understand help-seeking patterns among school-age adolescents in light of exposure to traumatic events. Based on this study’s findings, special attention should be paid to the impact certain events have on adolescents’ mental health and their help-seeking behaviours. Positive help-seeking behaviours that resonate with adolescents’ beliefs, emphasising contextual factors in mental health coping, should be promoted.

## 1. Introduction

Adverse childhood experiences (ACEs) are often used as an umbrella term for potentially traumatic events that could have negative long-lasting effects on health and wellbeing [1,2]. These events range from maltreatment, to various forms of abuse [1,3], to any event occurring before the age of 18 that causes harm or distress and consequently upsets one’s wellbeing [1,4]. In 2023, a systematic review by Madigan et al. estimated the prevalence of ACEs at around 60% in a population of 546,458 adults across 206 studies published between 1998 and 2021 from 22 countries [5]. Madigan et al. also estimated the mean prevalence of ACEs of 490,423 children aged 18 years or less at around 58% across 65 studies published between 1998 and 2024 from 18 countries [6]. However, the majority—if not all—of the included studies originated from European and North American countries with a negligible representation of the Eastern Mediterranean region (EMR) [5]. Hence, the need for primary research to establish a cross-study and multi-country distribution of these experiences in the region is essential.

It is well recognised that ACEs significantly influence the physical and mental wellbeing of an individual that has been linked to increased harmful behaviours and health risks [1,7]. These behaviours include increased smoking [8], substance use [9], obesity [10], chronic diseases [11,12,13], and adverse psychological conditions [8,14], among others [8,15]. Additionally, neglecting the inquiry of ACE and investment in appropriate prevention efforts comes at a great cost [7]. To illustrate, a systematic review by Hughes et al. reported that interpersonal violence exhibited the highest population-attributable fractions (PAFs) due to ACEs, ranging from 14.7% to 53.5% [7]. Also, the total ACE-attributable costs of consequent harmful outcomes ranged between USD 0.1 billion and USD 129.4 billion across 28 European countries with harmful alcohol use, smoking, and cancer incurring some of the highest costs [7].

When it comes to the young, struggles experienced secondary to suffering from a traumatic event during childhood result in higher support needs [16,17]. For instance, an online survey of 321 university students in the United States of America revealed that although having experienced an ACE increased the likelihood of seeking help, students were less likely to find interventions helpful, which led to higher unmet needs [16]. That is supported by similar recent studies from the United Kingdom (UK) showing that individuals who experience ACEs are constantly looking out for forms of emotional and practical support but largely hindered by a disappointing health service delivery [17,18]. This highlights that not only ACE shapes lifelong health but also the behaviours of help seeking [16,17,18].

Generally, research examining different aspects of ACEs as well as traumatic life events and associated help seeking is scarce [16,17,18] and almost non-existent in the EMR. This study consequently will add to the body of evidence and contribute to the knowledge gap of public health importance [16,17,18,19,20,21] considering that mental health morbidity is on the rise in Jordan [22,23,24,25] where young people (≤19 years) make up around 44% of the population [26]. Moreover, the country hosts the second-largest share of refugees per capita, 48% of whom are under the age of 18 [27]. Refugees are well known to have higher support needs and vulnerability to mental health disorders and related risk factors including ACE [20,23,28,29,30]. In general, adolescents tend to be particularly vulnerable to discrimination, stigma, social exclusion, educational difficulties, and risk-taking behaviours [31,32,33]; all are factors that inevitably influence help seeking.

This study aims to (1) estimate the prevalence of adverse events among adolescents in Jordan; (2) examine how traumatic life and adverse childhood events shape their mental health help-seeking behaviours; (3) investigate potential disparities in ACE and help seeking based on individual characteristics. This study provides an understanding of traumatic life events and ACE and its impact on health-seeking behaviour in this important population in the EMR, which is essential to guide targeted interventions aimed at mitigating their detrimental impact [16,17,18].

## 2. Materials and Methods

### 2.1. Participants

This study was conducted as part of a cross-sectional nationally representative survey of mental health and psychosocial problems among children and adolescents in Jordan [25,34]. The survey utilised a multi-stage stratified cluster sampling technique where representative coverage of basic and secondary schools in the Ministry of Education, and the private sector, and the United Nations Relief and Works Agency (UNRWA) for Palestine refugee schools in Jordan was achieved [25,34]. In this study, we solely focus on reporting the procedures and findings concerning the survey of adolescents living in Jordan including Jordanians and Syrian and Palestinian refugees (aged 12–18 years) undertaken between December 2022 and April 2023.

### 2.2. Instrumentation and Measures

Literature reviews and interdisciplinary expert consultations were sought for instrument selection and development [34]. We obtained all questionnaires from the developers and copyright holders, all of which are standardised, globally recognised, and validated in English [34]. The instruments were then culturally adapted and translated to Arabic using a forward–backward method [35]. All selected tools were then combined in one questionnaire and were pilot-tested. Further details on instrument adaptation and translation processes are highlighted in a separate study [25,34].

#### 2.2.1. Participants’ Characteristics

Family Affluence Scale (FAS)-III [36] was used to examine the socioeconomic status of the participants. FAS-III is a six-item measure of family wealth, which was slightly revised to a culturally and linguistically appropriate version in Arabic [36,37]. Additionally, a question about smoking status was also added specifically referring to conventional cigarettes or water pipe (shisha) use versus being a non-smoker. The response options for the questions varied depending on the type of question and included the following options and scores, no/not at all/none = 0, yes/one/once = 1, two/twice = 2, and more than twice = 3, which were totalled and scored between 0 and 13, with higher scores reflecting greater affluence [25]. Results were categorised for both boys and girls as follows: 0–7 indicating low affluence, 8–11 indicating medium affluence, and 12–13 indicating high affluence [25,38].

#### 2.2.2. Adverse Childhood Experiences (ACEs)

Childhood past exposure to an adverse or stressful event was assessed using two instruments: the Adverse Childhood Experiences (ACEs) [3] and the Life Events Checklist for DSM-5 (LEC-5) [39]. The ACE questionnaire is a 10-item questionnaire measuring adverse childhood experiences before age 18 and is derived from the Centre for Disease Control and Prevention (CDC)-Kaiser Permanente Adverse Childhood Experiences Study [3]. It reflects 10 types of potential childhood trauma, which were revised by the expert panel where—due to cultural sensitivity—certain original items, such as those on sexual abuse and family-related issues, were excluded. Responses were binary (“happened to me” or “did not happen to me”). The other tool was the Life Events Checklist for DSM-5 (LEC-5) [39], which aims to assess exposure to 16 events known to potentially result in Post-Traumatic Stress Disorder or distress and includes one additional item assessing any other extraordinarily stressful event not captured in the first 16 items. In this study, only 11 questions from LCE-5 were added to complement the ACE questionnaire, along with three new items describing trauma related to exam failure and COVID-19. Responses were limited to two choices: (happened to me) or (did not happen to me). Permission to use the measure was obtained from the National Center for PTSD at the United States Department of Veterans Affairs.

#### 2.2.3. Intention of Help Seeking—The General Help-Seeking Questionnaire (GHSQ)-Revised

The intention to seek help when facing an adverse event was assessed using the General Help-Seeking Questionnaire (GHSQ) [40]. It is a two-item questionnaire designed to assess the intent to seek help, capturing different sources and reasons for help seeking. The original version contains two questions on intentions to seek help and sources of help for general personal and emotional problems and suicidal ideation. The GHSQ was revised and modified to fit our study aims and contextual settings [25,34]. A screening question, “*If you had a psychological problem, would you seek help?*”, with binary responses (yes, no) was included while questions pertaining to suicidal thoughts were omitted as they did not align with the aims of the study. Additionally, the sources of help were adjusted to reflect the known sources of help among adolescents in Jordan, based on the expert panel recommendations [25].

### 2.3. Pilot Testing

Pilot testing was undertaken in two schools in Northern Jordan on 7 November 2022 for questionnaire content evaluation [25,34]. A total of 30 students (15 from each school) participated in the pilot, which helped assess readability, and clarity, and identify logistical problems. Changes identified were considered and reflected in the modification of the questionnaires in both languages.

### 2.4. Data Analysis

Data from the surveys were combined in one dataset and were analysed by using IBM SPSS 24 (IBM Corp. Released 2016. IBM SPSS Statistics for Windows, Version 24.0. Armonk, NY, USA: IBM Corp). Categorical variables were described as frequencies and percentages. Proportions were compared using the Chi-square test. Binary logistic regression was used to determine the factors associated with the adolescents’ intention to seek help in case of an adverse event. *p*-Values of 0.05 or less were considered statistically significant.

## 3. Results

A total of 4407 adolescents aged between 12 and 18 years were included, the majority of which were females (55.5%) and attended public schools (63.8%). Jordanians represented the largest proportion of the sample at 66.2%, followed by Syrians residing outside the refugee camps at 16.8%. Approximately 51.1% of adolescents lived in the central governorates of Jordan. In contrast, 30% resided in the northern regions, and only 7% were located in the southern governorates. Around three-quarters reported that their fathers were currently working, and 25% reported that their mothers were currently working where about half (55.5%) fell within the medium Family Affluence Scale. Around 28.3% of adolescents reported having previously been infected with COVID-19 (Table 1).

### 3.1. The Adverse Childhood Experiences

Prevalence estimates of the four categories of ACEs (1–4 ≥ ACEs) in our sample population are as shown in Table 2. The estimated prevalence of four or more ACEs is around 43% in contrast to only around 12% of no events.

The most reported ACEs among adolescents (Table 3) were being infected or having a family member infected with COVID-19 (60.3%), failing an exam (43.2%), and the sudden accidental death of any family member (31.7%). On the other hand, the least reported experiences were assault with a weapon (6.3%) and exposure to toxic substances (8.3%).

### 3.2. Adverse Childhood Events and Intention of Help Seeking

The intention of help seeking was significantly influenced by exposure to three particular ACEs: exposure to fire or explosion, being infected or having a family member infected with COVID-19, and feeling that they are not beloved by their families (Table 4). Adolescents who reported having been exposed to fire or an explosion were less likely to seek help in comparison to non-exposed adolescents (15.7% vs. 20.5%, *p* = 0.005) as well as those who feel that they are not beloved by their families (17.2% vs. 20.5%, *p* = 0.030). However, adolescents who have been infected or had a family member infected with COVID-19 were more likely to seek help (22.1% vs. 16.1%, *p* < 0.001).

### 3.3. Individual Characteristics’ Impact on the Likelihood of Help Seeking

Certain individual characteristics and negative childhood experiences showed influence over the likelihood of adolescents’ help-seeking intention (Table 5. Medium and high family affluence were significantly associated with decreased odds of seeking help compared to the low-affluence group (OR: 0.65, 0.70, *p*-value: <0.001, 0.005, respectively). Smokers had a significantly decreased likelihood of seeking help compared to non-smokers (OR: 0.63, *p*-value: <0.001). Amongst the adverse events, having been exposed to fire or explosion was significantly associated with decreased odds of help-seeking intentions (OR: 0.72, *p*-value: 0.021) while being infected themselves or having a family member infected with COVID-19 was significantly associated with increased odds of help-seeking intentions (OR: 1.50, *p*-value: <0.001). The model was adjusted for other variables including age and gender.

## 4. Discussion

Childhood adversity is a determinant of health [41] to which tailored interventions could have substantial health and economic benefits for affected populations [7]. Positive help-seeking behaviours are key for effective use of services to combat the deleterious impact of such adversities [7,42]. Responding to an urgent call [31] for local research to investigate help-seeking behaviours among adolescents experiencing mental health issues in Jordan, this study aimed to examine the prevalence of ACEs and traumatic life events among adolescents and their impact on help-seeking patterns.

In a recent study of the same population [25], it was revealed that when facing a problem, only around one-fifth (19.7%) of adolescents would seek help. The majority also preferred informal sources of help such as their parents or friends (81% and 60.2%, respectively), over professional sources, e.g., doctors (49.8%) and mental health professionals (47.3%), and school-based sources, i.e., counsellors and schoolteachers (31.5% and 34.8%, respectively). That is particularly concerning in light of the high prevalence of ACEs reported in our study.

### 4.1. The Prevalence of Adverse Events

Around 88% of our study population have experienced one or more ACEs in their lifetime. Specifically, slightly less than half (43%) have experienced four or more ACEs in their lifetime. That is around 28% more than the estimated average prevalence of this category of ACEs (mean prevalence: 14.8%, 95% CI: 5.1–24.8) in the same age group globally [6]. Additionally, the most reported ACE by our population was COVID-19 (60.3%).

COVID-19 was first detected in Jordan in March 2020 [43] where as of April 2023, there has been 1,746,997 confirmed cases and 14,122 deaths in Jordan [44], one of the highest recorded numbers of deaths among the Arab countries [45]. Justifiably, our results showed being infected or having a family member infected with COVID-19 to be the most commonly reported ACE (60.3%) among adolescents. COVID-19 has been shown to be significantly detrimental for mental health [46,47,48,49]. Salameh et al.’s systematic review on COVID-19’s impact on mental health showed an increased prevalence of PTSD; psychological disorders; symptoms of anxiety, stress, and depression; and symptoms of mental ill-health among different population groups in Jordan [46]. Additionally, COVID-19 has been associated with an increased number of reported ACEs among adolescents, in line with our findings [50,51]. Such an increase been linked to the impact of lockdown and subsequent social and educational disruptions on individuals’ mental health globally [43,50,51,52]. Post-pandemic impact, however, is thought to be diverse, as although most would have been exposed to COVID-19 and its challenges, not all recover the same or recover at all [53]. Our study answers a heightened call amongst the scientific community to consider COVID-19 in itself as an adverse event rather than an exacerbating factor [53,54], in that in almost 33 months after the first reported case in Jordan, our results show that COVID-19 impact as an adverse event is predominantly significant.

The second most reported ACE among participants was failing an exam (43.2%). Adolescent students in Jordan often include those in their final two years of high school gearing towards college entrance examinations, which adds to the tremendous fear of potential failure and high parental expectations, all of which are likely injurious to their wellbeing [55,56,57]. A recent systematic review [32] including studies from 1991 to 2022 has shown that academic pressure is positively correlated with multiple mental health disorders including depression, anxiety, and self-harm. This study, however, posed a limitation as few studies originated from low- and lower–middle-income countries and none from the Middle East. Locally, a cross-sectional study [57] of 405 public high school female students reported that academic challenges and lower GPA were significant predictors for depression and anxiety, which aligns with our findings and previous global research [56,58,59,60,61,62].

### 4.2. Adverse Events and Intention to Seek Help

Evidence is lacking on the impact of specific adversities on help-seeking behaviours. Adolescents in our study were 4.8% less likely to seek help had they been exposed to fire or an explosion than the non-exposed adolescents (15.7% vs. 20.5%). The impacts of disasters including fires and explosive violence, including the potential loss of safe spaces, are reportedly linked to mental instability and disorders, e.g., PTSD, anxiety, and depression [63,64,65,66]. One cross-sectional survey [67] conducted in light of the 2020 Beirut explosion examined the help-seeking pattern of 2078 individuals aged between 18 and 60 and above and reported that about two-thirds of the sample (62.2%) did not seek any help following the explosion, 26.3% sought non-professional help, and only 11.5% sought professional help, which aligns with our findings that are in itself concerning since this group is potentially vulnerable with great need for professional help.

Families and trusted relationships appear to play a critical role in shaping adolescents’ behaviour towards seeking help for mental health issues [68,69,70]. As previously reported [25], less than 20% of adolescents in our cohort would seek help for psychological problems from informal sources of help, preferred over professional or school-based sources. Arab countries in particular enjoy a unique context and different conceptualization of psychological problems [71], where people are more reliant on and trusting of informal sources owing to their accessibility and lower associated stigma [72,73,74,75,76]. Moreover, the family unit and the surrounding society play a pivotal role in shaping adolescents’ viewpoints, their understanding of adversity, and consequently their help-seeking behaviour [72,73,74,75,76]. Understandably, then, adolescents in our study who felt that they were not loved by their families were 3.3% less intent on seeking help for their psychological problems (17.2% vs. 20.5%). Although feeling loved is subjective and complex to interpret, the lack of family support is a known barrier to adolescents seeking help [68,77,78].

On the contrary, adolescents who have been infected/have had a family member infected with COVID-19 showed more willingness to seek help than their counterparts (22.1% vs. 16.1%). Since data before the pandemic in Jordan are lacking, no correlation could be made on whether this figure reflects a higher awareness of mental health issues brought about by the pandemic as highlighted by past research that the level of literacy about a given disease could potentially impact individuals’ help-seeking behaviour [68,79,80,81,82,83]. In light of the pandemic, a nationwide prospective cohort study in Germany of 648 adolescents investigated the change over time in adolescent’s attitude pre-lockdown and after the first lockdown in March 2020 [84]. Their study revealed that, compared to the pre-lockdown sample, participants reported more positive attitudes towards seeking professional help for a mental health problem [84]. In that regard, our study is adding to the literature reliant on a representative study sample.

### 4.3. Characteristic Differences and the Likelihood of Help Seeking

Adolescents from medium and high family affluence in our study were less likely to seek help for psychological problems. Both poverty and affluence come with their own adversities and attitudes towards help seeking [85]. A recent review of 19 studies, including 1 from the EMR, found that youth from low and medium affluence used health services more than those from higher affluence [85,86]. This may be due to the culture of privacy in affluent families, especially when parents hold powerful societal positions [85,87].

Interestingly, adolescent smokers in our study reported significantly decreased odds of seeking help (OR: 0.63, CI: 0.50–0.80, *p*: <0.001). Synonymously, a cross-sectional study [88] conducted in 2006 in Malaysia of 399 students revealed that although smokers reported higher emotional and behavioural challenges, 94.7% of them did not seek professional help for their problems. As smoking in itself has been associated with poor mental health as well as overall wellbeing and vice versa [88,89,90,91], the relationship between smoking in adolescence and decreased intention for help is worrying and needs to be further examined.

### 4.4. Implications

It is vital to highlight that determinants of help-seeking behaviours are complex and multifactorial in nature where elements of anticipated social and psychological costs, adolescent–healthcare provider relationship dynamics, and personal and contextual traits, among others, could influence the likelihood of seeking help in the case of facing an adversity [92,93,94]. In our cohort, for example, the most reported reason of not seeking help largely centred around religiosity where 73.7% of the adolescents stated that it is God’s will and they resign themselves to their state as fate [25]. Religiosity is an integral part of the majority of Arab societies [95,96,97], similarly for Jordan, which is predominantly a Muslim country at 97.1% of the population [98]. Research on the relationship between religiosity and help-seeking attitudes is scarce and often shows mixed results [95,96,99,100].

That shows the importance of unravelling factors at play in shaping help-seeking behaviours, particularly considering the prevalence of adversities and largely negative attitudes towards seeking help, so that interventions resonate with the religious and cultural beliefs, among other factors, driving adolescents’ help seeking.

## 5. Conclusions

Despite extensive evidence linking adverse events to poor health outcomes, little is known about help-seeking attitudes among adolescents, especially in the Eastern Mediterranean. Our study revealed significant prevalence levels of adverse events among adolescents in Jordan. That is concerning given the predominant negative perception of help seeking. Specific adverse events and socioeconomic characteristics influence the likelihood of professional help seeking for mental health support more than others, namely, exposure to fire/explosion or feeling unloved by family, family affluence, and smoking. Our findings suggest that help-seeking behaviour is influenced by the type of adversity faced as well as the contextual factors of the population. Hence, it is important for policymakers to prioritise and implement context- and adversity-sensitive interventions for this age group. Future research into potential mediators of the relationship between ACEs and help seeking could provide valuable guidance for target intervention planning.

## Figures and Tables

**Table 1:** Sociodemographic Characteristics and Health Status of adolescents.

Sociodemographic Characteristics	N (4407)	%
**School classification**		
Public school	2811	63.8
Private school	717	16.3
UNRWA school	343	7.8
Non-formal education centre	86	2.0
Zaatari Camp School	450	10.2
**School type**		
Male	1716	38.9
Female	1801	40.9
Mixed	890	20.2
**Gender**		
Male	1959	44.5
Female	2448	55.5
**Region**		
Middle	2254	51.1
South	449	10.2
North	1704	38.7
**Nationality**		
Jordanian	2917	66.2
Syrian living in a camp	510	11.6
Syrian living outside the camps	739	16.8
Palestinian living in a camp	76	1.7
Other	165	3.7
**Parents’ marital status**		
Living together	3938	89.4
Separated	233	5.3
Either parent deceased	236	5.4
**Mother education**		
Diploma or higher education	1666	37.8
Less than diploma	2741	62.2
**Father education**		
Diploma or higher education	1630	37.0
Less than diploma	2777	63.0
**Working father**	3465	78.6
**Working mother**	1101	25.0
**Family income source**		
The work of one or both parents	3732	84.7
Humanitarian organisations	212	4.8
Relatives and friends	80	1.8
Having no income	383	8.7
**Monthly income (JD)**		
Less than 300	1725	39.1
301 to 500	1391	31.6
>500	1291	29.3
**Have health insurance**	2360	53.6
**Family affluence**		
Low affluence	723	16.4
Medium affluence	2446	55.5
High affluence	1238	28.1
**Ever had COVID-19**		
Yes *	1247	28.3
Unsure	1028	23.3
No	2132	48.4

* Confirmed by a positive test or based on medical advice.

**Table 2:** Prevalence of Adverse Childhood Experiences Among Adolescents.

No. of Adverse Events	n	%
No events	543	12.32
1 ACE	648	14.70
2 ACEs	692	15.70
3 ACEs	615	13.96
4 or more ACEs	1909	43.32

**Table 4:** Differences in Intention of Help seeking According to Adverse Childhood Events.

Adverse Childhood Event	Intention of Help Seeking				
	No		Yes		*p*-Value
	n	%	n	%	
**Natural disaster**					0.966
Did not happen to me	2904	80.3	712	19.7	
Happened to me	357	80.2	88	19.8	
**Fire or explosion**					0.005
Did not happen to me	2726	79.5	702	20.5	
Happened to me	538	84.3	100	15.7	
**Car accident**					0.058
Did not happen to me	2372	81.1	553	18.9	
Happened to me	881	78.5	242	21.5	
**Serious accidents at home or school or during recreational activity**					0.134
Did not happen to me	2741	80.0	687	20.0	
Happened to me	520	82.5	110	17.5	
**Exposure to toxic substance**					0.809
Did not happen to me	2983	80.3	730	19.7	
Happened to me	275	80.9	65	19.1	
**Physical assault**					0.312
Did not happen to me	2492	80.0	622	20.0	
Happened to me	759	81.5	172	18.5	
**Assault with a weapon**					0.642
Did not happen to me	3049	80.3	748	19.7	
Happened to me	207	81.5	47	18.5	
**Combat or exposure to a warzone**					0.967
Did not happen to me	2838	80.4	694	19.6	
Happened to me	411	80.3	101	19.7	
**Life-threatening illness or injury**					0.751
Did not happen to me	2871	80.2	709	19.8	
Happened to me	375	80.8	89	19.2	
**Sudden accidental death of any family member**					0.486
Did not happen to me	2224	80.6	535	19.4	
Happened to me	1023	79.7	261	20.3	
**Infected or a family member infected with COVID-19**					<0.001
Did not happen to me	1348	83.9	258	16.1	
Happened to me	1898	77.9	539	22.1	
**A family member died from COVID-19**					0.441
Did not happen to me	2895	80.4	704	19.6	
Happened to me	332	78.9	89	21.1	
**Serious injury or harm you caused to someone else**					0.106
Did not happen to me	2743	79.9	690	20.1	
Happened to me	472	82.8	98	17.2	
**Insulted or belittled by an adult in the house**					0.729
Did not happen to me	2509	80.4	613	19.6	
Happened to me	733	79.8	185	20.2	
**Severely beaten by an adult in the house**					0.053
Did not happen to me	2705	79.8	685	20.2	
Happened to me	527	83.1	107	16.9	
**Feeling that no one in your family loves you**					0.030
Did not happen to me	2496	79.5	644	20.5	
Happened to me	744	82.8	155	17.2	
**Did not have enough to eat, or had to wear old clothes**					0.274
Did not happen to me	2741	79.9	689	20.1	
Happened to me	504	81.8	112	18.2	
**Failing an exam**					0.287
Did not happen to me	1869	79.6	478	20.4	
Happened to me	1384	81.0	325	19.0	

**Table 5:** Factors Hindering the Likelihood of Help seeking.

	OR	95% CI		*p*-Value
Family affluence				
Low affluence	1.00			
Medium affluence	0.65	0.52	0.82	<0.001
High affluence	0.70	0.54	0.90	0.005
Smoking	0.63	0.50	0.80	<0.001
**Adverse childhood events**				
Natural disaster	1.13	0.84	1.53	0.411
Fire or explosion	0.72	0.55	0.95	0.021
Car accident	1.21	0.99	1.47	0.058
Serious accidents at home or school or during recreational activity	0.93	0.72	1.21	0.595
Exposure to toxic substances	1.21	0.85	1.71	0.284
Physical assault	0.98	0.77	1.24	0.855
Assault with a weapon	1.04	0.67	1.60	0.874
Combat or exposure to a warzone	1.23	0.92	1.64	0.166
Life-threatening illness or injury	0.97	0.72	1.32	0.868
Sudden accidental death of any family member	1.02	0.84	1.23	0.852
Infected or a family member infected with COVID-19	1.50	1.24	1.81	<0.001
A family member died from COVID-19	0.97	0.72	1.32	0.856
Serious injury or harm you caused to someone else	0.77	0.58	1.03	0.077
Insulted or belittled by an adult in the house	1.22	0.96	1.57	0.108
Severely beaten by an adult in the house	0.80	0.59	1.08	0.138
Feeling that no one in your family loves you	0.81	0.63	1.04	0.099
Did not have enough to eat, or had to wear old clothes	0.97	0.74	1.28	0.838
Failing an exam	0.96	0.80	1.16	0.697

**Table 3:** Adverse Childhood Experiences Among Adolescents by type.

Reported Adverse Childhood Events	n	%
Natural disaster	472	10.8
Fire or explosion	670	15.4
Car accident	1215	28.0
Serious accidents at home or school or during recreational activity	674	15.5
Exposure to toxic substances	361	8.3
Physical assault	1004	23.2
Assault with a weapon	273	6.3
Combat or exposure to a warzone	542	12.5
Life-threatening illness or injury	489	11.3
Sudden accidental death of any family member	1373	31.7
Infected or a family member infected with COVID-19	2611	60.3
A family member died from COVID-19	454	10.5
Serious injury or harm you caused to someone else	615	14.3
Insulted or belittled by a parent or other adult in the house	992	22.9
Severely beaten by a parent or other adult in the house	694	16.1
Feeling that no one in your family loves you	953	22.0
Did not have enough to eat or had to wear old clothes	654	15.1
Failing an exam	1878	43.2

## Data Availability

Restrictions apply to the availability of these data. Data were obtained from The Eastern Mediterranean Public Health Network (EMPHNET) and will be made available by the authors upon reasonable request conditional on permission from EMPHNET.
